# Diseases of the Breast

**Published:** 1888-06

**Authors:** 


*Diseases of the Breast.* By THOMAS BRYANT, F.R.C.S. Pp. 358. London: CASSELL & CO. 1887.

We have become so much accustomed to a high degree of excellence in Cassell's Clinical Manuals, that we always expect pleasure and profit from the perusal of each new volume. This one is no exception to the rule, and in many respects is better than most of its fellows. The work is sound, practical, and up to date; it is clearly and simply written; and it is illustrated with original and well-executed lithographs and engravings. While the work is fully representative of the prevalent modern opinions of the best authorities, it is also in many respects distinctly personal. A special word of praise is merited for the clear arrangement and accurate division of the chapters, sections, and paragraphs.

